# Patients’ experiences of living with Long Covid and their beliefs about the role of psychology in their condition

**DOI:** 10.1177/13591053251325112

**Published:** 2025-03-17

**Authors:** Saara Petker, Jane Ogden

**Affiliations:** University of Surrey, UK

**Keywords:** beliefs, Long Covid, psychology, trust, uncertainty

## Abstract

Some patients with chronic conditions are unreceptive to a psychological approach. This study aimed to explore the experience of Long Covid (LC) with an emphasis on patients’ beliefs about the role of psychology. UK participants (*n* = 14) with either self-reported or diagnosed LC took part in semi-structured interviews. Thematic analysis described three main themes: ‘Living in uncertainty’, ‘Why should I trust you if you don’t believe me?’ and ‘Once I know the cause people will believe me’. Transcending these themes was a tension between professional experts and experts by lived experience and a dichotomy between psychological and medical explanations. Overarching all themes was a sense that synthesising the biological and psychological components of LC could help to resolve this tension. In summary. living with LC is characterised by feeling disbelieved which can drive the rejection of psychology. Helping patients feel listened to may encourage a more positive approach to psychological support.

## Introduction

Following the Covid-19 pandemic, an increasing number of infected individuals reported experiencing ongoing symptoms for weeks, months and even years after initially falling ill (National Health Service [NHS] England, 2020). This came to be known as ‘Long Covid’, a term generated by patients who shared their experiences with each other online of this novel, uncertain and debilitating condition (Callard and Perego, 2021).

Symptoms of Long Covid are wide-ranging, with the most common including fatigue, cognitive difficulties, breathlessness and muscle pain (Office for National Statistics [ONS], 2023); The National Institute for Health and Care Excellence [NICE], 2022). While there is still no internationally recognised definition of Long Covid, NICE guidelines propose that the acute Covid infection transitions into Long Covid when symptoms continue to persist for 4 weeks post-infection (NICE, 2022). Specifically, symptoms lasting between 4 and 12 weeks after illness onset can be classified as ‘ongoing symptomatic Covid-19’, whilst symptoms lasting 12 weeks are known as ‘post Covid-19 syndrome’ (NICE, 2022). Worldwide, estimates indicate that approximately 6%–10% of adults are affected by Long Covid following initial infection (Zheng et al., 2023) and that up to 46.5% report not being fully recovered by 12 months with the most common symptoms being fatigue/malaise, headache, shortness of breath and muscle/joint pain (Pazukhina et al., 2024). Data estimates that as of January 2023, there are currently around 2 million people in the UK living with Long Covid (ONS, 2023) compared with 1.3 million in January 2022 (ONS, 2023). Whilst Long Covid seems to effect individuals regardless of the severity of their original infection (Yong, 2021) there is evidence that Long Covid is more prevalent in females (ONS, 2023) those of increasing age, white ethnicity and poorer pre-pandemic general and mental health (Thompson et al., 2022). It is also more prevalent in Higher Income Countries although its impact on the individual may be greater in Lower and Middle Income countries (Pazukhina et al., 2024)

Despite growing attention, the aetiology, diagnosis and treatment of Long Covid remains unclear. There has, however, been a surge in research exploring the biological mechanisms underpinning persistent symptoms with findings suggesting an inflammatory response, a continuing presence of the virus remaining in the body, or reinfection of a different strain amongst an array of other medical hypotheses (Davis et al., 2023; Tay et al., 2020). These findings remain speculative, however, and are yet to inform a direction for biomedical treatment. Consequently, evolving research has started to take a biopsychosocial approach to understanding Long Covid as a long-term condition (Hussain, 2022). A large body of existing literature illustrates the role of psychological factors in long-term conditions comparable to Long Covid, such as chronic fatigue (Hempel et al., 2008; Hulme et al., 2017), Rheumatoid Arthritis (Geenen and Dures, 2019), irritable bowel syndrome (Windgassen et al., 2017) and chronic pain (Giusti et al., 2021; Hruschak and Cochran, 2018). These studies posit that while biological processes play a role in illness onset, psychological factors, such as coping style, interpretation of illness and sense making or perceived social support will influence how illness is experienced and the impact it has on an individual’s daily life and draw upon health psychology theoretical frameworks such as the self-regulatory model of illness (SRM; Leventhal et al., 1980, 2016).

Whilst researchers have widely adopted a biopsychosocial approach to chronic conditions, not all patient groups are equally enthusiastic (Geraghty et al., 2019; Komaroff, 2015; Sharpe and Greco, 2019; Tesio and Buzzoni, 2021). This has been particularly apparent in those patients living with Chronic Fatigue Syndrome (CFS/ME; Geraghty et al., 2019; Komaroff, 2015). Whilst the disease pathology for CFS/ME remains unclear, CFS/ME patient organisations have been active in opposing the emphasis on psychological factors for their condition due to the collective experience that this approach downplays the seriousness of their illness and leads to the neglect of biomedical research (Blease and Geraghty, 2018; Geraghty et al., 2019; Komaroff, 2015). Strong views put forward by this patient group around the management of their condition led NICE to delay and amend its planned publication of its updated guidelines for the diagnosis and management of CFS/ME in 2021 (NICE, 2021). Furthermore, pushback from the CFS/ME patient groups led to the removal of Cognitive Behavioural Therapy (CBT) as a treatment for CFS/ME reflecting the evolving power of the patient voice and a cultural shift towards patient-informed care.

Evidently, it is important that Long Covid patients are invited to share their experience of illness and their views on treatment involving psychological intervention. Existing literature has justifiably focussed on gathering objective data on aetiology and effective treatments for Long Covid which are integral to the development of clinical management plans. However, for these initiatives to be effective and utilised by patients with Long Covid it is important that they are informed both by objective findings as well as the subjective patient experience of living with Long Covid (Ladds et al., 2020). The synthesis of objective and subjective findings would enhance current understandings of how to best meet the physical, psychological and social needs of this patient group. A qualitative exploration is particularly important given the strong patient voice and sharing of personal experiences in the development of Long Covid as a recognised illness. Moreover, it is essential to obtain a deeper understanding of the narratives surrounding psychology and Long Covid and explore potential barriers between this patient group and psychological input.

A rapidly emerging area of research has started to explore Long Covid from the perspective of those experiencing it. A recent systematic review and meta-synthesis of 15 qualitative studies investigating the experience of living with Long Covid provided a rich overview of the subjective patient perspective (Hossain et al., 2023). The review produced six major synthesised findings including the physical symptoms of Long Covid, the psychosocial impact of Long Covid, the long and slow recovery, the need for more patient information, changes in social support and challenges with the healthcare system. Of these, one study by Burton et al. (2022) focussed specifically on the psychological aspects of Long Covid and concluded that feeling heard and validated by professionals and loved ones were important factors for psychological well-being. It similarly noted that poor mental wellbeing can have a negative impact on physical symptoms such as breathlessness and fatigue. Whilst the findings from Burton et al.’s (2022) study provide some insight into the psychological effects of living with Long Covid, the focus was specifically on mental health rather than the broader psychological processes included in a health psychology perspective with its focus on sense making, coping and adaptation (e.g. Leventhal et al., 1980, 2016). Similarly, existing research has not explored patient attitudes towards psychological thinking regarding Long Covid. This is a key feature of the present study, as understanding barriers to psychology is inherent in informing patient-focussed services that will be utilised by Long Covid patients. Further, with the continually growing number of patients developing Long Covid and our understanding of treatments evolving, it is important to continue to capture their subjective experiences to compliment emerging biomedical research. In line with previous research the present study therefore aimed to explore the broader psychological experience of Long Covid with a particular focus on Long Covid patients’ attitudes towards psychology and its role in their condition.

## Method

### Design

This study used a qualitative semi-structured interview design to gather rich and detailed experiences of Long Covid. Data was analysed using reflexive thematic analysis (Braun and Clarke, 2012). An inductive approach was chosen given the limited literature available on the topic area which drew upon a critical realist epistemological perspective allowing the researcher to consider the data as a reflection of the participants’ objective realities whilst also acknowledging that these realities each sit within a wider cultural, societal, and historical context (Bhaskar, 2010). The study received favourable approval from the University Ethics Committee (FHMS 21-22 282 EGA) on 01/11/2022.

### Participants

The inclusion criteria were adults aged 18 or over with Long Covid defined as symptoms of Covid-19 lasting longer than the acute 4 week period of initial infection. Participants did not require a formal Long Covid diagnosis or a previous positive Covid-19 test confirming prior infection. Fourteen UK-based participants took part in the interview, consisting of 12 females and two males aged 27–63 (median = 47 years). Eleven participants identified as White British, one as White Irish, one as Asian British (Indian) and one as mixed-ethnic background (Indian and Portuguese). Eleven participants were recruited through a previous study during which they left their contact details for follow-up. Three further participants were recruited via the researcher’s social network to broaden the participant. See Table 1 below for a full list of participants.

**Table 1.** Description of participants.

Pseudonym	Age	Ethnicity
Emma	39	White British
Poppy	63	White British
Kerry	28	White British
Louise	61	White British
Sophie	37	White British
Greg	30	White British
Sarah	51	White British
Nicole	47	White British
Holly	52	White British
Sameera	27	Asian British
Jo	57	White British
Danny	52	Mixed Ethnic Background
Cynthia	42	White Irish
Janet	47	White British

### Interview schedule

The interview schedule was as follows: ‘Can you tell me your experience of having Long Covid?’; ‘Can you tell me about the impact your illness had/is having on your life?’; ‘Can you tell me why you think your symptoms carried on?’; ‘What kinds of things you have done to try and make things better for yourself?’; ‘What do you think, from your own perspective, might help you manage your illness or recover?’; ‘What’s your understanding or view of how the way in which we think, feel or behave can impact on illness?’. Interviews took place between April 2022 and March 2023, lasted 25–35 minutes and were transcribed before undergoing analysis.

### Data analysis

Inductive reflexive thematic analysis was used to examine responses from the interviews in line with Braun and Clarke (2012): familiarisation with the data set by reading and re-reading the transcripts several times; assignment of codes to summarise items of interest; identification of overlapping or related codes; re-examination of codes to refine themes; re-reading data to check consistency of themes; and finally, defining themes and translating them into a meaningful and unified narrative. The lead author carried out the first level of coding which was then discussed and changed through both authors examining transcripts and discussing the best way to frame the analysis. In keeping with the reflexive element of thematic analysis, reflective discussions with the research supervisor and a reflexive summary helped the researcher to actively engage in reflection on biases which may have influenced their interpretation of the data.

### Positionality

Both researchers are psychologists. The lead author who also carried out the interviews was in training as a clinical psychologist. The second author was her supervisor and an academic psychologist. Both therefore brought with them an interest and endorsement of psychological approaches. Both had had COVID-19 but neither had experienced Long Covid. Both identified as female. These factors may have influenced our engagement with the participants and our interpretation of the data. To mitigate against this, we discussed our own biases at length and how we felt about the possibility of psychological approaches as helpful or unhelpful for those with physical health conditions. We also ensured that we kept re examining the data to remain close to the participants’ own experiences.

## Results

**Figure 1.**
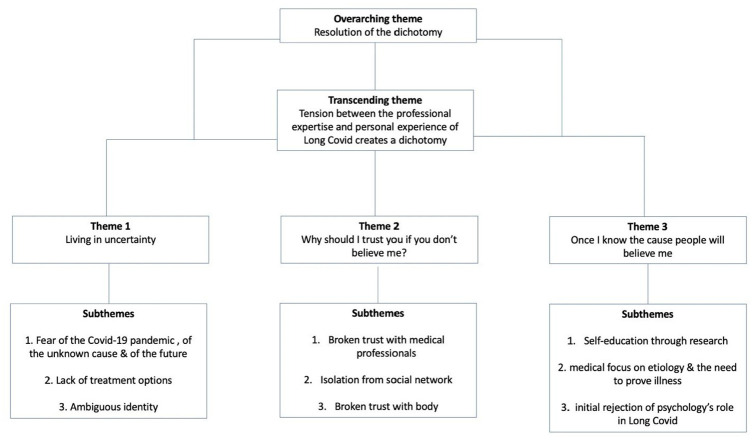
Thematic map.

The analysis produced three main themes with subthemes: ‘Living in uncertainty’; ‘Why should I trust you if you don’t believe me?’ and ‘Once I know the cause, people will believe me’. Transcending these themes was a tension between the professional experts of Long Covid and the experts by lived experience of the condition, and a consequent dichotomy between the psychological and physical explanations of Long Covid. Overarching these themes was the resolution of this dichotomy through an acceptance of the synthesis of the medical and psychological components of Long Covid. A thematic map can be seen in Figure 1. These themes with exemplar quotes are described below.

### Theme 1: Living in uncertainty

Participants described living with Long Covid as characterised by uncertainty in terms of: fear of Covid-19, lack of treatment options and ambiguous identity.

#### Fear of covid-19

Participants spoke about the portrayal of the pandemic in the media and the fear that this evoked during their Long Covid illness. For example, Jo described feeling unnerved by her environment at a time when she was seeking containment and comfort:

Every time you turned on the, the television, or you had the news, or the sirens going past the road, that was a big part of it actually … it sort of made me feel … well, worse. It … perhaps it took the hope out of you know the … that reassurance. (Jo)

Many participants described a fear of the unknown cause of Long Covid. For example, Greg described a sense of fear and frustration that there is still little understanding of Long Covid’s cause and trajectory and therefore little progress on a recovery plan for patients:

It’s scary from a medical world standpoint, what’s going on and how we are not getting on top of all this. (Greg)

Participants consequently described the uncertain future of living with Long Covid as frightening. For example, Greg captured this sense of fear:

…what’s so scary is kind of like … the uncertainty of like what my future is going to look like. (Greg)

#### Lack of treatment options

Participants shared a sense of uncertainty pertaining to the lack of treatment options available for Long Covid. For example, Emma described how access to Long Covid treatment was dependent on location and expertise available within that area, emphasising the lack of widely available, standardised Long Covid treatment:

I have been seen by a Long Covid clinic and been dismissed because I don’t fit their … the particular symptoms that that department looks at where I am in my catchment area. (Emma)

Another participant, Louise, who met the criteria for a Long Covid service described her experience of inadequate treatment options within the service:

It took nearly a year for my referrals to the Long Covid clinic to actually happen … there was no treatment, it was just online assessments, a questionnaire assessment talk with somebody and then a series of six online sessions … talking through symptoms and how to manage them. It was all stuff I’d heard before because it had taken me a year to get to that point. (Louise)

Louise described issues with lengthy waiting lists for Long Covid services and shared the perspective that guided self-management of Long Covid symptoms as a standalone treatment was inadequate. She communicated a sense of disappointment and unease regarding the treatment options on offer.

#### Ambiguous identity

Participants reflected on their identities pre and post Long Covid, making comparisons between their past, present and future selves. For example, Sameera described how Long Covid had changed her character and her ability to engage with her friends in the same way as she had before:

I’m not the same person I was kind of last year. I might not be able to celebrate [at my friend’s wedding] in a way I would normally be able to because of my energy levels. (Sameera)

Sameera clearly described the impact Long Covid had on her ability to carry out her usual role of supporting her friend. Some participants described an uncertain identity coupled with a clearer sense of loss of identity. For example, Poppy described this sense of loss in relation to her role at home and how she used to be more able to look after others:

Mentally [I’m] very down. Sometimes very, very weepy, very weak just because it’s not me. I know it’s not me … You know where I used to make lunch and supper, I can just make supper now. (Poppy)

Poppy described feeling detached from her sense of self and expressed a transition of roles from being the provider to the one who is provided for.

Participants therefore described a sense of living with uncertainty in terms of fear, lack of treatment options and ambiguity over their identity.

### Theme 2: Why should I trust you if you don’t believe me?

In theme two, participants documented experiences of feeling disbelieved, unheard, and dismissed which resulted in a breakdown of trust. This was described in terms of a loss of trust in medical experts, social networks and their own bodies.

#### Broken trust with medical experts

Participants described seeking reassurance about their illness from medical experts but failing to obtain this due to the lack of available medical knowledge. For example, Sarah explained how the lack of medical reassurance impacted her ability to cope with her illness:

The fact that experts don’t really know what’s going on, the fact that I don’t feel I was listened to, that had a profound effect on how I coped with it [Long Covid]. I think if there had been more help at the beginning and someone [with expertise] had said, ‘yeah what’s happening basically is this, this is how we’re gonna try and deal with it’, we don’t know, but I think it would have had a more positive effect. I might have accepted things more and coped better. (Sarah)

A lack of medical reassurance led Sarah to feel unheard and uncontained by the experts. She explained how she would have wanted her doctor to demonstrate their expertise and collaborate with her, conveying the message that she instead felt dismissed and left to understand and manage her illness alone. She expressed feeling let down by the experts who were unable to uphold their usual role of providing her with a medical explanation and treatment plan, and perhaps more importantly, did not demonstrate an intention to listen, investigate or support her further, breaking Sarah’s trust. Emma shared similar sentiments in feeling dismissed and went further to describe feeling disbelieved by her medical professionals:

I have taken what they [medical professionals] are saying to me as them saying ‘this is all in your head’ and I felt terrible about that and invalidated and like I’m doing something wrong and it’s my fault. (Emma)

Emma clearly described the impact of being told that her symptoms did not have a known cause. She voiced a strong sense of feeling invalidated, disbelieved, and at fault for her condition.

#### Isolation from social network

Participants also reported experiences of their Long Covid illness being disbelieved and poorly understood by employers, friends and family members, ultimately leading to a breakdown in trust in these relationships and increased social isolation. For example, Louise conveyed the invisibility of Long Covid as an illness which made it difficult for others to observe how debilitating the condition can be, creating issues with belief from others:

Occupational health did not know a thing, and weren’t really believing me, my employer definitely wasn’t, friends are getting it a bit more now but don’t really get it because they see you on good days. (Louise)

Sameera described how having Long Covid created a sense of isolation from her wider social circle. At times this was due to not feeling believed but at others it related to a sense of not being understood:

No one I know has had the same experience as me, so it can feel quite isolating … I have supportive friends … but they’re not in my position, they don’t fully understand how I might be feeling. (Sameera)

#### Broken trust with body

Participants described losing trust in their bodies after developing Long Covid. The unpredictability of symptoms and lack of control over their impact were highlighted as the main causes of this distrust. For example, Jo described her anxieties about her relationship with her body:

I didn’t trust my body … I didn’t know what was happening to it and I didn’t feel like I could manage it on my own (Jo)

Jo described a loss of expertise about her body. Nicole similarly explained an incongruence between her bodily symptoms and her medical test results. She expressed a loss of trust with her body, as it no longer collaborated with Nicole and failed to provide medical results which would enable others to believe her illness:

I was still feeling pretty rubbish … went to the GP … had all sorts of things [tests] as you can imagine - you know ECGs and heart scans and lung scans and CT scans, you know, just everything. And you know pints of blood taken from me. And obviously they all come back completely negative … and gradually as the time has gone on … additional symptoms would come up. (Nicole)

Participants therefore described how the invisibility and uncertainty of Long Covid led to disbelief from others, resulting in a broken sense of trust in medical experts, a feeling of isolation from social networks, and a loss of trust in their own bodies.

### Theme 3: once I know the cause, people will believe me

Theme three described how participants felt pressured into adopting a medical position to explain their Long Covid illness, as this seemed to be the only way to persuade others to believe their condition. Participants turned to self-education of Long Covid through research of medical literature in search of a physical cause of Long Covid. The focus on the medical aetiology of their illness and the need to prove their condition led to the rejection of the psychological components of Long Covid.

#### Self-education through research

Participants described a process of relying less on their medical experts and turning to their own resources to research a physical cause of their condition. For example, Sarah depicted a need to regain control of investigating her condition, as the medical professionals were unable to do so:

I wanted help from primary sources like my GP but they didn’t wanna know and so I just have to do it on my own … I think it’s got to be self-guided. (Sarah)

Louise described reading medical journals in her own research of Long Covid’s aetiology and drawing on medical hypotheses to make sense of her condition:

I was reading lots of research including medical journals I don’t necessarily understand but try to understand, looking for hope really … (Louise)

Louise explained how researching medical answers instilled a hope that overcoming Long Covid was a possibility.

#### Medical focus on aetiology and the need to prove illness

Participants proposed several hypotheses for Long Covid’s aetiology which maintained strong medical stances. For example, Emma explained her palpitations with the belief that Long Covid had altered her cardiac function:

I have thousands and thousands of extra beats per day which is what gives me the sense of palpitations. (Emma)

Sophie described how she thought her symptoms were due to an inflammatory immune response:

It felt to me like inflammation in the body, as if my body was being attacked. (Sophie)

Louise shared how her medical test results proved to others that her illness was indeed an organic issue and invalidated the narrative that her illness was a construct of her mind:

I had a 24-hour ECG which picked up some tachycardia, so something definitely was going on, it wasn’t just in my head as everyone likes to say. (Louise)

#### Initial rejection of psychology’s role in Long Covid

Participants described a perception of Long Covid’s aetiology being either physical or psychological, and feeling forced into adopting the medical position (as this enabled others to believe their condition) which ultimately led to a rejection of psychological understandings of Long Covid. Some participants conveyed this rejection implicitly, for example, Louise described an acceptance of taking antidepressant medication for her physical symptoms of Long Covid but not necessarily for low mood or anxiety brought on by living with Long Covid:

I’ve been put on sertraline by my GP which is an SSRI but not for anxiety or depression, but they’re finding it can help people’s fatigue. (Louise)

Jo similarly conveyed an implicit dismissal of psychology and attributed changes in her psychological state during her illness to an organic and physical change brought on by Long Covid:

I was so emotional at that stage; I think it [Long Covid] had hit my whole sort of autonomic nervous system. (Jo)

Other participants portray a more explicit rejection of psychological thinking around Long Covid. For example, Poppy described believing that her condition was physical in causation and had no psychological component:

I don’t see how you could do the psychological in the sense that I know I’ve done a couple of hours work, and I know I’m done now cognitively – that’s not psychological. Because actually I want to carry on working, I’m frustrated the fact that I can’t do anything, so I don’t think that that’s a mental, that’s, that’s a physical thing. (Poppy)

Poppy clearly described a rejection of the idea that her symptoms are ‘psychological’ in aetiology and likened this idea to being told that her illness is all in the mind or ‘mental’. Participants therefore described how they attempted to take control of their recovery by researching and proving the organic aetiology of their Long Covid, in turn rejecting psychological models of Long Covid.

### Transcending theme: Tension between medical experts versus experts by lived experience of Long Covid

Participant psychological experiences of Long Covid can therefore be understood in terms of three main themes relating to: living in uncertainty, feeling disbelieved by others which leads to a loss of trust and social isolation, and finally searching for a cause in a bid to help others believe their condition and access the medical care they require. However, efforts to seek a cause did not provide satisfactory outcomes, as no medical answer for Long Covid was available. Therefore, transcending these three major themes was a frustration amongst participants regarding their unmet need for a medical cause of Long Covid and a treatment plan. These frustrations were fuelled by a perceived sense of powerless with medical experts and rising tensions between those who were experts of Long Covid by profession and those by lived experience. This tension resulted in a perceived dichotomy between psychological and medical models of Long Covid’s process.

Greg depicted a tension between the two expert groups (Long Covid patients and medical experts) and highlighted the ‘us versus them’ narrative:

I’ve been told by the doctor that this [Long Covid] is in my head … but if you ask me … this is my, it’s my lived experience, and then I’m seeing that this narrative is being then, you know, reinforced through my parents … and then other long haulers out there, and I find it really actually quite insulting when I’ve got people out there who suddenly say like … you know, you can think yourself out of there [illness] or you’re being lazy. (Greg)

Greg’s quote illustrated the contradiction of beliefs about Long Covid between those who were medically trained experts of the condition and those who became experts through living with Long Covid. He conveyed frustrations around the collective struggle and failure for Long Covid patients to have their medical needs met despite their attempts to seek and prove the organic cause of their illness. Participants described how their frustrations were fuelled by power imbalances between them and their medical professionals. For example, Emma explained how she felt silenced and overpowered by medics due to the hierarchical structure of patient-doctor relationship dynamics despite feeling that she was the expert of her own Long Covid experience:

…a lot of people living with [Long Covid] are now finding that they know far more than the medical professionals they’re seeing because they’re keeping up with the research where the medical professionals can’t … uh cause they’re too busy … which is enormously frustrating … because you’re also being treated like you’re not the expert and actually you are on a fairly level pegging in some of these circumstances. (Emma)

Participants described a need to regain personal control as a consequence of feeling powerless; however, this reinforced that there was no medical cure for Long Covid and maintained the cycle of frustration. For example, Poppy illustrated her need for controlling her illness with medical intervention and the resulting frustration that followed:

I take vitamin D, I do vitamin C. I breathe in iodine. So, all the things that general people are saying, ‘Oh, that could help’, I’ve done all of that. I rest and it doesn’t get any better. (Poppy)

Participants therefore described how the focus on a search for a cause proved to be unsuccessful in producing satisfactory outcomes and intensified tensions between medical professionals and Long Covid patients. It also deepened the dichotomy between the physical and psychological models of Long Covid.

### Overarching theme: Finding resolution in the dichotomy through a shift from ‘what’s the cause?’ to ‘how do I cope?’

Finally, overarching all transcripts was the process by which some participants resolved their frustrations through an acceptance of the current unavailability of a clear medical answer for Long Covid. Participants described a shift from focussing their attention on a cause and cure for their condition towards living and coping with it. Such participants’ transcripts illustrated how this had helped them regain a sense of control by exercising choice over how they responded to and self-managed their illness. Through this process, participants shifted from rejecting psychological thinking altogether to beginning to demonstrate an initial acknowledgement of its role in Long Covid management, particularly in helping participants live with their condition. In doing so, a resolution of tensions between Long Covid patients and medical professionals followed, as participants began to recognise the synthesis between the medical and psychological models in Long Covid management.

For example, Emma described how she had begun to recognise the role of psychological support for Long Covid in the absence of an identifiable biological cause:

With hindsight I don’t actually think that’s what they were saying to me [‘the illness is all in your head’] but that is how it comes across. And I think it is really difficult if you haven’t got any, like physical, medical treatments to offer it makes sense that you’re offering psychological support because all that you can do in that situation is live with it. (Emma)

Likewise, Nicole made a reference to the mind-body interaction and described how she moved towards a recognition of the impact that her behaviours, thoughts, and emotions had on her experience of Long Covid:

Now I’ve come to terms with well, it’s just happened, we are where we are … improvements physically have happened because I have changed my mindset on it I think … I have stopped looking at things like that [blood oxygen monitor], because they make me feel more anxious and more concerned and more frightened about what’s happening to my body and when that happens the symptoms get worse. (Nicole)

Jo also described an explicit acknowledgement of the biology- psychology synthesis in Long Covid management, sharing the view that her recovery would not rest solely on medical intervention:

You have to look at all sides of it, your physical health, but your mental health, all of it, the whole lot. It has to be all joined up, not, there’s no one thing … there’s certainly no one pill or medical thing that’s gonna solve it. (Jo)

Participants therefore described a resolution of the psycho-medical dichotomy through an initial recognition of the synthesis of these models in Long Covid management.

## Discussion

The present study aimed to explore the broader psychological experience of Long Covid with a particular focus on patients’ attitudes towards psychology and its role in their condition.

In summary, the analysis described three themes: ‘Living in uncertainty’, ‘Why should I trust you if you don’t believe me?’ and ‘Once I know the cause people will believe me’. Transcending these themes was a tension between Long Covid patients and healthcare professionals, and a dichotomy between medical and psychological explanations of Long Covid. Finally, overarching these themes was a resolution of the dichotomy and patient-doctor tensions through an acceptance of the synthesis between medical and psychological perspectives on how Long Covid is experienced.

The first theme described the uncertain experience of living with Long Covid in relation to fear, a lack of available treatment options, and ambiguous identity. These findings are consistent with existing qualitative research which documents fear of Covid-19 infection, reinfection and infecting others as a common emotional experience for Long Covid patients (Hossain et al., 2023), as well as fear about the timeline of Long Covid illness and recovery (Day, 2022). The findings also suggest that uncertain treatment plans may create feelings of helplessness for Long Covid patients in relation to managing their condition and obtaining a sense of certainty over their illness. Kingstone et al. (2020) found that Long Covid patients felt that the NHS Long Covid recovery advice to ‘pace’ activities was unhelpful and led to feelings of helplessness. The final subtheme of ambiguous identity is also reflected in existing literature on identity in chronic illness, as Long Covid patients appear to experience a dispossession of social roles and valued identities, creating a sense of loss and grief in relation to their identity as a healthy person (Ahlström, 2007; Charmaz, 1994; Lindgren, 1996).

The second theme described a loss of trust in healthcare professionals, social networks and their own body for people with Long Covid. Consistent with existing research, numerous participants in the present study described the collective struggle of obtaining medical reassurance and having their illness validated as ‘real’ by experts (Ireson et al., 2022; Kingstone et al., 2020). The findings imply that feeling believed by medical professionals is equally important as receiving medical advice in reducing illness-related distress and maintaining patient trust (Kingstone et al., 2020). This reflects previous research which proposes the idea that the ‘good’ clinician is not merely a diagnostician but is also an active-listener and professional witness (Heath, 1998; Ladds et al., 2020). Feeling disbelieved by employers, friends and family led to similar breakdowns in trust but with additional experiences of social isolation and perceived loneliness, consistent with existing research (Burton et al., 2022). This finding suggests that Long Covid patients endure the consequences of living with an invisible illness and face injustice in medical and societal care when compared to more visible conditions (Ireson et al., 2022). In accordance with research on chronic and poorly explained conditions, the present findings suggest that Long Covid patients experience higher levels of psychological distress, social isolation and perceived loneliness than those with medically well-explained conditions (Dirkzwager and Verhaak, 2007). These findings highlight the significant impact that feeling believed and understood by others can have on the experience of Long Covid.

The third theme described how participants coped with their illness by searching for a physical cause to prove that their Long Covid is ‘real’. This finding can be understood in relation to Leventhal et al.’s self regulatory model (Leventhal et al., 1980, 2016). Participants appeared to appraise their former coping strategy of seeking medical reassurance from professionals as ineffective because of the lack of identifiable disease and often feeling dismissed. Instead, they adopted the alternative coping strategy of self-directed information-seeking, due to the belief or hope that they may find a physical cause which would solve the problem of Long Covid. This mirrors the longstanding experiences of patients with CFS/ME and other medically unexplained conditions (Ireson et al., 2022), whereby the biomedical approach, which is central to the medical model, leads healthcare professionals to conclude that the absence of identifiable organic pathology means there is no real illness (Bayliss et al., 2014). Consequently, patients are cornered into adopting a medical position to prove the realness of their condition and work towards a cure (Werner and Malterud, 2003). The findings showed that proving illness entails a long and time-consuming process of negotiating with healthcare services for further medical tests and scans and engaging in self-directed medical research. Such behaviours can heighten attention on symptoms and leave less time for potentially useful clinical interactions. Further, the attempt to prove the biological aetiology of Long Covid indirectly leads to a rejection of psychological thinking around the condition.

Transcending the three main themes was a tension between the experts by lived experience (Long Covid patients) and experts by profession (healthcare professionals), resulting in a dichotomy between medical and psychological models of Long Covid’s process. For example, self-directed research led to little advancement in Long Covid management due to power dynamics at play during patient-doctor interactions. That is, patients’ testimonies of their lived experience and own research were disregarded on the basis that the medical expert ‘knew better’, resulting in patient frustrations fuelled by feelings of powerlessness. Furthermore, doctors’ perspectives that their illness had no clear biological pathology and therefore must be psychological in nature, appeared to feed the narrative that their illness was ‘all in the mind’, intensifying the dichotomy between the two models. These findings reflect the large body of existing literature on the ongoing contest between biological and psychosocial models for conditions such as CFS/ME, fibromyalgia and chronic pain syndromes (Sharpe and Greco, 2019; Tesio and Buzzoni, 2021), and the ‘splitting’ of medicine. Similarly, research on epistemic injustice in Long Covid (Ireson et al., 2022), fibromyalgia (Heggen and Berg, 2021) and chronic pain (Buchman et al., 2017) has also documented patient testimonies being discredited by medical professionals, highlighting the experience of powerlessness in patients with invisible illnesses like Long Covid.

Overarching these themes was a resolution of the dichotomy through a recognition of the synthesis between medical and psychological mechanisms underpinning Long Covid. In line with this, some participants recognised a role for both biology (for onset) and psychology (for management) in their illness experience (Sharpe and Greco, 2019). This process of moving from dichotomy to resolution can be understood in terms of the notion of cognitive dissonance and the patient’s search for coherence (Festinger, 1954; Leventhal et al., 1980, 2016). Cognitive dissonance theory posits that when two cognitions are inconsistent with each other dissonance occurs, creating psychological discomfort which in turn motivates the individual to reduce dissonance and search for coherence (Festinger, 1954). Long Covid patients experience cognitive dissonance when they receive information that there is no pathology for their illness, thereby querying the reality of their illness. Patients seek coherence by avoiding information that increases dissonance (Harmon-Jones and Mills, 2019), leading to the initial rejection of psychological thinking. However, this results in a polarisation of biological and psychological perspectives and increased cognitive dissonance. Patients do not alter their cognitions to suggest that Long Covid has a psychological aetiology, as such a cognition would be more resistant to change because it does not align with their reality (i.e. the experience of physical symptoms) and thus would maintain cognitive dissonance. Instead, patients adopt a cognition that is consistent with their reality. Some, therefore, start to believe in a biological cause to their problem whilst starting to accept that psychology can have a role to play - not in terms of onset, but in the course and intensity of their experience through psychological processes such as thoughts, feelings, and behaviours. Therefore, whilst patients with Long Covid reasonably reject claims that psychological processes play a causal role in their illness, they may be more receptive to the suggestion that psychology has a place in the management of Long Covid as this helps them resolve their dissonance and find coherence.

There are some limitations that need to be considered. First, participants self-reported their diagnosis of Long Covid and many did not have a confirmed diagnosis of COVID-19. This was in part due to the timing of the interviews (April 2022 and March 2023) allowing the researcher to capture lived experiences of Long Covid as awareness and understanding of the condition evolved. However, this also means that the patient group in the current study may not reflect those who currently have a more formal diagnosis of Long Covid. Further, the sample was predominantly White and female, which although reflects the population most affected by Long Covid, may not accurately reflect the lived experiences of wider populations. Furthermore, given the focus on psychology both in terms of the disciplines of the researchers and the research focus, the recruitment process may have resulted in a sample more open to psychological thinking. Finally, given that the interviewer was a psychologist and the research focus was on psychology, participants may have felt more predisposed to speak more positively about psychology due to issues of social desirability.

The findings from the present study have several implications for clinical practice. First, they highlight the need for Long Covid patients to feel believed and supported by their health professionals. To achieve this, health professionals could share their own uncertainties around the causes of Long Covid rather than dismiss the patient’s symptoms as having no organic cause (Chew-Graham et al., 2017; Kingstone et al., 2020). Further, they could use a more collaborative approach which recognises the patient’s expertise through lived experience which can promote feelings of validation and empowerment (Gorske and Smith, 2008). In addition, health professionals could also play a role in educating the patients support networks to support Long Covid patients in feeling validated and heard to prevent social withdrawal. Secondly, the findings inform how patients can be supported psychologically. Whilst patients desire a biological cause to their condition, they may be more open to accepting psychological support if they can see benefits for their daily experiences of symptoms. Psychological interventions that help patients manage their symptoms could include a focus on increasing tolerance of uncertainty, reducing feelings of helplessness, rebuilding an identity outside of Long Covid, psychoeducation on the mind-body interaction and increasing social engagement. These proposed psychological interventions reflect NICE guidelines for psychological input in the management of Long Covid (NICE, 2021). Finally, the findings illustrate how health professionals need to offer psychological interventions alongside medical support rather than as a substitute for it as a means reduce the mind body dichotomy and help patients find coherence between their own experiences of physical symptoms and the psychological support available. Together these clinical steps could help shift the narrative that Long Covid is ‘all in the mind’ and build more positive relationships between Long Covid patients and psychological support.

In summary, the present qualitative study aimed to explore how patient with Long Covid experience their condition and their attitudes towards psychology. The analysis described three themes relating to uncertainty, trust and the need to be believed which were transcended by a tension between professional experts and experts by lived experience and a consequent dichotomy between the psychological and medical explanations of Long Covid. For some patients, this tenson could be resolved, however, by accepting a synthesis of the biological and psychological components of Long Covid and acknowledging that whilst the search for a biological cause can continue, psychology may have a role to play in the management of their daily lives. Accordingly, if health professionals want to effectively support patients with Long Covid, they need to make sure they feel believed and offer psychology as an adjunct rather than a substitute for medical support, as a means to move on from the outdated and harmful narrative of it being ‘all in the mind’.
